# Regulation of Innate Lymphoid Cells in Acute Kidney Injury: Crosstalk between Cannabidiol and GILZ

**DOI:** 10.1155/2020/6056373

**Published:** 2020-02-25

**Authors:** Babak Baban, Hesam Khodadadi, Kumar Vaibhav, Cristina Marchetti, Carlo Riccardi, Mahmood S. Mozaffari

**Affiliations:** ^1^Department of Oral Biology and Diagnostic Sciences, The Dental College of Georgia, Augusta University, Augusta, Georgia 30912-1128; ^2^Department of Neurosurgery, Medical College of Georgia, Augusta University, Augusta, Georgia 30912-1128; ^3^Department of Medicine, University of Perugia, Italy

## Abstract

Innate lymphoid cells (ILCs) have emerged as largely tissue-resident archetypal cells of the immune system. We tested the hypotheses that renal ischemia-reperfusion injury (IRI) is a contributing factor to polarization of ILCs and that glucocorticoid-induced leucine zipper (GILZ) and cannabidiol regulate them in this condition. Mice subjected to unilateral renal IRI were treated with the following agents before restoration of renal blood flow: cannabidiol, DMSO, transactivator of transcription- (TAT-) GILZ, or the TAT peptide. Thereafter, kidney cells were prepared for flow cytometry analyses. Sham kidneys treated with either cannabidiol or TAT-GILZ displayed similar frequencies of each subset of ILCs compared to DMSO or TAT, respectively. Renal IRI increased ILC1s and ILC3s but reduced ILC2s compared to the sham group. Cannabidiol or TAT-GILZ treatment of IRI kidneys reversed this pattern as evidenced by reduced ILC1s and ILC3s but increased ILC2s compared to their DMSO- or TAT-treated counterparts. While TAT-GILZ treatment did not significantly affect cells positive for cannabinoid receptors subtype 2 (CB2+), cannabidiol treatment increased frequency of both CB2+ and GILZ-positive (GILZ+) cells of IRI kidneys. Subsequent studies showed that IRI reduced GILZ+ subsets of ILCs, an effect less marked for ILC2s. Treatment with cannabidiol increased frequencies of each subset of GILZ+ ILCs, but the effect was more marked for ILC2s. Indeed, cannabidiol treatment increased CB2+ GILZ+ ILC2s. Collectively, the results indicate that both cannabidiol and GILZ regulate ILC frequency and phenotype, in acute kidney injury, and that the effects of cannabidiol likely relate to modulation of endogenous GILZ.

## 1. Introduction

Dysregulation of immune and inflammatory mechanisms contributes importantly to pathogenesis of a variety of disorders including acute kidney injury (AKI) [1–3]. Consequently, better understanding of involved mechanisms and identification of novel targets of therapy have been the mainstay of research in the field.

Glucocorticoids are well-recognized for their widespread effects on multiple organ systems and for their regulation of diverse physiological functions which contribute to maintenance of basal- and stress-related hemostasis. Therapeutically, glucocorticoids have been used for diverse disorders with the common denominator of dysregulation of immune and inflammatory mechanisms [4]. Nonetheless, recognition of their adverse metabolic effects has prompted unraveling of mechanisms which contribute to their prominent anti-inflammatory effects. These studies have provided strong evidence in favor of glucocorticoid-induced leucine zipper (GILZ) protein as the pivotal regulator of anti-inflammatory effects of glucocorticoids [5–7]. Thus, therapeutic GILZ has raised the prospect of curtailing pathogenic inflammation while avoiding adverse metabolic effects of glucocorticoids.

Aside from glucocorticoids, the endocannabinoid system has emerged as another endogenous pathway regulating immune and inflammatory mechanisms, among other functions [8–14]. While the use of cannabis dates back to ancient history, the discovery of *Δ*9-tetrahydrocannabinol, as the major psychoactive constituent of cannabis, led to a flurry of research which ultimately established that the endocannabinoid system is comprised of endogenous ligands (e.g., anandamide) and the biochemical machinery responsible for their synthesis, transport, and degradation as well as specific cannabinoid receptors (i.e., CB1 and CB2); while CB1 receptors are primarily located in the central nervous system, immune cells primarily express CB2 [8]. Nonetheless, both cannabinoid receptor-dependent and receptor-independent effects have been described for cannabinoids [8]. More recently, cannabidiol has received much attention as a potential therapeutic agent as exemplified by its recent approval, by the Food and Drug Administration, for treatment of seizures associated with two rare and severe forms of epilepsy [15].

We recently showed that both GILZ and cannabidiol exert renoprotective effects, as reflected by reduction in cell death and improved functional outcomes, in the murine model of AKI simulated by ischemia-reperfusion injury (IRI); the renoprotection of GILZ and cannabidiol was accompanied with generally similar effects of each agent on polarization of neutrophils and T lymphocytes [16, 17]. In light of these observations, we decided to explore potential impact of cannabidiol and GILZ on innate lymphoid cells (ILCs) in AKI.

ILCs are largely tissue-resident immune cells which respond early in the course of immune response (compared to T lymphocytes); they are uniquely positioned to respond quickly to pathogens and other environmental stimuli to maintain homeostasis. ILCs are divided into 3 classes (i.e., ILC1s-ILC3s) depending on their cytokine signaling requirement and transcription factors for their lineage commitment [18–21]. ILC1s-ILC3s lack adaptive antigen receptors and are functionally considered innate counterparts of T lymphocytes, mirroring CD4+ T helper (Th)1, Th2, and Th17 cells, respectively [18–21]. In mice, and under the influence of cytokine signaling and certain transcription factor(s), innate lymphoid cell precursors differentiate to give rise to each subset of ILCs; ILC1s utilize T-box transcription factor (T-bet), ILC2s express GATA binding protein 3 (GATA3) and depend on RAR-related orphan receptor *α* (ROR*α*) for their activity and development, while ILC3s require transcription factor RAR-related orphan receptor *γ*t (ROR*γ*t) and aryl hydrocarbon receptor (AhR) for their activity and development [18, 19]. Functionally, ILCs are characterized by their cytokine profile and their participation in various immune responses. For example, ILC1s produce interferon *γ* (IFN*γ*) and mediate type 1 immunity while ILC2s produce interleukin- (IL-) 4, IL-5, IL-9, and IL-13 and mediate type 2 immunity, and ILC3s produce IL-17 and IL-22 and mediate type 3 immunity [18, 19]. Despite their important roles in immune responses against pathogens/injurious stimuli and in the maintenance of tissue homeostasis, uncontrolled and differential activation of ILCs may elicit inflammatory responses that further result in pathological conditions. Importantly, similar to T cells, ILCs demonstrate a certain degree of plasticity thereby allowing them to change their phenotype and functional capacities depending on environmental cues such as polarizing signals in the tissue along with the expression of cognate cytokine receptors and key transcription factors in responding ILCs [19–21]. This property likely contributes to the important roles of ILCs not only in immune responses against pathogens and sterile inflammation but also in maintaining tissue hemostasis. Nonetheless, differential development and activation of ILCs in a context- and/or disease-specific manner may contribute to inflammatory responses and/or outcome of treatment modalities [20–27].

In light of the above, we tested the hypothesis that renal IRI is a contributing factor to polarization of ILCs and that GILZ and cannabidiol regulate them in this condition. Further, in light of evidence indicating an interaction between glucocorticoids and endocannabinoid signaling pathways [8], we determined whether an interaction exists between cannabidiol and GILZ in regulation of ILC subtypes in AKI.

## 2. Materials and Methods

### 2.1. Animals

The studies utilized male, 9-11 weeks of age, Balb/C mice which were obtained from Harlan Laboratories and housed in the laboratory animal facilities of the Augusta University with free access to food and water. Rodents of similar age are routinely used for the investigation of various pathologies and the use of male mice relates to greater susceptibility of the male gender to AKI [28]. These studies conformed to guidelines of Institutional Animal Care and Use Committee.

### 2.2. TAT and TAT-GILZ

The protocol for generation of the transactivator of transcription (TAT) peptide and the TAT-GILZ fusion protein has been described previously [29]. Briefly, TAT and TAT-GILZ, which was constructed by inserting GILZ cDNA in the TAT C vector to produce an in-frame fusion protein, were cloned into the pGEX-4T2 plasmid (GE Healthcare). The pGEX-4T2 plasmid is a glutathione S-transferase (GST) fusion vector carrying a tac promoter for chemically inducible high-level expression of the protein. GST fusion protein was expressed in lipopolysaccharide- (LPS-) lacking bacteria Clear Coli BL21 (Lucigen Corporation, Middleton, USA) which were grown at 37°C and induced with 1 mM isopropyl b-D-thiogalactopyranoside for 4 hours [30]; all other materials used in the process were sterile and LPS-free. Following sonication to induce lysis, most of the generated protein was found in the soluble fraction, which was then purified with Glutathione Sepharose 4B beads (GE Healthcare) following the manufacturer's instructions. Eluted proteins were dialyzed against PBS for 48 hrs.

### 2.3. Renal IRI and Treatment Protocols

Animals were anesthetized with ketamine (120 mg/kg, i.p.)/xylazine (16 mg/kg, i.p.) in preparation for the surgical procedure which consisted of two flank incisions and clamping of the left renal pellicle, for 20 min, using a nontraumatic vascular clamp while the right kidney served as sham control. Thereafter, the vascular clamp was removed and restoration of renal blood flow confirmed, visually, prior to closure of muscle and skin layers using 4-0 silk sutures and autoclips, respectively [16, 17]; each independent experiment included 3-6 animals, and the number of animals for each group/condition is indicated under Figure legends. Each of the following agents was administered, intraperitoneally in a volume of 50 *μ*l, 10 min before restoration of renal blood flow: (a) vehicle (DMSO), (b) cannabidiol (10 mg/kg, 16), (c) TAT (0.1 mg/kg in PBS), and (d) TAT-GILZ (0.2 mg/kg in PBS); the latter dosage regimen is based on a two-fold larger molecular weight of GST-TAT-GILZ than GST-TAT as well as studies indicating resolution of LPS-induced inflammation in response to TAT-GILZ treatment and its effectiveness in the murine model of acute kidney injury [17, 29]. Postoperative analgesia was provided with a single injection of buprenorphine (1 mg/kg, SC). The animals were sacrificed 1-day postinjury and kidneys procured for cell preparation and flow cytometry-based assays.

### 2.4. Flow Cytometry Analyses

For flow cytometry analysis, renal tissues were sieved through a cell strainer (BD Biosciences, San Diego, CA), followed by centrifugation (1,500 rpm, 5 min) to prepare single-cell suspensions. Preparative cell sorts were performed on cells stained with fluorochrome-conjugated monoclonal antibodies using a 4-Laser LSR II flow cytometer as well as a Coulter MoFlo XDP cell sorter [20]. Cells were gated based on forward and side scatter properties and on marker combinations to select cells of interest [20]. Total murine ILCs were gated as Lineage^−^CD45^+^ lymphocytes (lineage cocktail of antibodies included PerCP-conjugated anti-CD3, anti-CD4, anti-CD14, anti-CD16, anti-CD19, anti-CD8, anti-CD15, anti-CD20, all from BioLegend) to exclude non-ILCs. Subsequently, ILC1s were identified as Lineage^−^CD45^+^CD127^+/-^IL-12R*β*2^+^ cells, ILC2s as Lineage^−^CD45^+^CD127^+^GATA3^+^ cells, and ILC3s as Lineage^−^CD45^+^CD127^+^ ROR*γ*t^+^ cells (all antibodies from BioLegend). The capacity to produce cytokines for ILC1s (IFN-*γ*/TNF-*α*), ILC2s (IL-5/IL-13), and ILC3s (IL-17/IL-22) was tested, as described previously [20]. Further, GILZ expression was assessed in ILCs subset using anti-GILZ antibody following similar flow cytometry protocol. In addition, whole-kidney cell preparations were also subjected to flow cytometry analyses for detection of ILC2s followed by further analyses to assess those positive for expression of CB2 and GILZ using anti-CB2 (Bioss, Cat no. BS-2377R-A488) and anti-GILZ (eBioScience, Cat no. 12-4033-80) antibodies. Isotype-matched controls were analyzed in each sample to set the appropriate gates; representative panels are reported in relevant Figures. For each marker, samples were analyzed with duplicate measurements.

### 2.5. Sorting of ILC2s, Cytospin Preparation, and Immunofluorescent Staining

Preparative cell sorts to obtain ILC2s were performed on kidney cells stained with fluorochrome-conjugated mAbs (sources as detailed, below) using the BD Biosciences FACSCAria II SORP equipped with 4 lasers and 18 fluorescence detectors. Kidney cells were gated based on forward and side scatter properties and on marker combinations to select cells of interest [20]. Total ILCs were gated as Lineage^−^CD45^+^CD127^+^ lymphocytes. The lineage cocktail of antibodies included FITC-conjugated anti-CD3, anti-CD4, anti-CD14, anti-CD16, anti-CD19, anti-CD8, anti-CD15, anti-CD20, anti-CD33, anti-CD34, anti-CD203 (eBioscience), and anti-Fc*ε*RI (BioLegend), with CD127 and Sca-1—both PerCP-conjugated (BioLegend). Subsequently, ILC2s were sorted as Lineage^−^ cells positive for GATA-3.

Cytospin preparations of ~10000 sorted cells per sample chamber were centrifuged (700 r.p.m., 5 min), air-dried, fixed in 10% formalin, and washed twice in PBS. All subsequent procedures were carried out in the dark and at room temperature. Endogenous peroxidase activity was blocked with hydrogen peroxide (1 : 10 w/PBS, 10 min). Immunofluorescence (GILZ and CB2) for cytospin preparations was stained as above. To permeabilize, all preparations were incubated in 0.2% Triton X-100 for 5 min at room temperature. All slides were washed three times for 5 min at room temperature and then incubated in blocking buffer (20% normal donkey serum, 1% BSA, 0.02% NaN3, 13 PBS) for 45–60 min. All slides were incubated with anti-CB2 (Bioss, Cat# BS-2377R-A488) and anti-GILZ (eBioScience, Cat# 12-4033-80) antibodies for 2 hours in the dark at room temperature. All slides were washed and then counterstained using DAPI (Thermo Fisher Scientific, D3571).

### 2.6. Statistics

Data were analyzed using the analysis of variance followed by Newman–Keuls post hoc test to establish significance (*p* < 0.05) among multiple groups. Statistical comparison of two groups (i.e., CB2+ GILZ+ ILC2s data) utilized Student's *t*-test. Data are reported as means ± SEM.

## 3. Results

**Figure 1:**
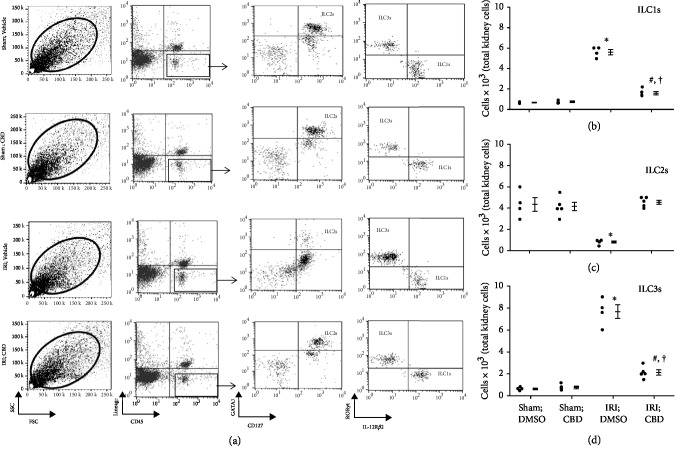
Effects of cannabidiol on ILCs subsets in the murine model of acute kidney injury. Mice were subjected to left renal ischemia- (20 min) reperfusion (1 day) injury (IRI) while the right kidney served as sham control; the animals were subdivided to receive intraperitoneal administration of either vehicle (DMSO) or cannabidiol (CBD), as described under Methods, before restoration of renal blood flow. Thereafter, cell preparations of kidneys from each animal were subjected to flow cytometry analyses for determination of ILC subsets using specific markers. (a) Gating strategy based on side scatter (SSC) vs. forward scatter (FSC) of kidney cells from experimental groups followed by identification of each subset of ILCs relying on phenotypic markers as described in Methods. (b–d) Quantitative data for each ILC subtype for each experimental group; data are individual values as well as means ± SEM of *n* = 4‐5 animals/group/condition; each independent experiment included 3-5 animals. ^∗^*p* < 0.05 compared to other three groups. ^#^*p* < 0.05 compared to their vehicle-treated counterparts. ^†^*p* < 0.05 compared to sham-operated groups.

Figure 1(a) depicts gating strategy based on side scatter vs. forward scatter of kidney cells from experimental groups followed by identification of each subset of ILCs relying on phenotypic markers as described in Methods. Figures 1(b)–1(d) show quantitative data for each ILC subtype for each experimental group. Sham-operated kidneys showed similar frequencies of each subtype of ILCs. However, IRI was associated with significant increases in ILC1s and ILC3s but a marked reduction in ILC2s (Figures 1(b)–1(d)). Treatment of IRI kidneys with cannabidiol reversed this pattern as indicated by significant reductions in ILC1s and ILC3s but increased ILC2s. However, while ILC1s and ILC3s of cannabidiol-treated IRI kidneys remained higher than sham-operated kidneys, no difference was noted in ILC2s between cannabidiol-treated IRI kidneys and sham-operated kidneys (Figures 1(b)–1(d)).

**Figure 2:**
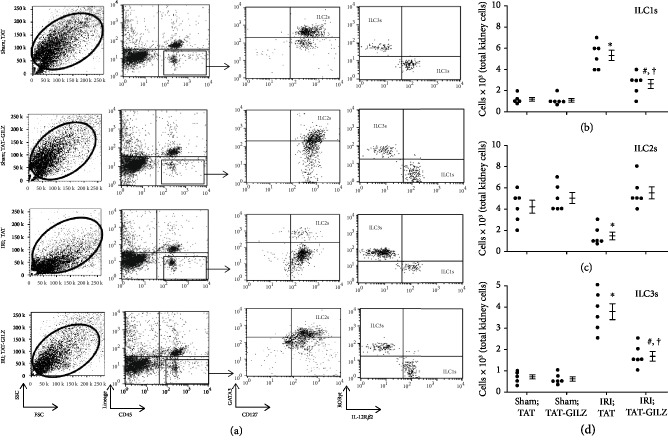
Effects of GILZ on ILC subsets in the murine model of acute kidney injury. Mice were subjected to renal IRI or sham operation and treated with either TAT or TAT-GILZ followed by assessment of subsets of ILCs in the kidneys as described under Figure 1 legend (representative flow cytometry data is shown under (a)). (b–d) Individual values and means ± SEM of *n* = 6 animals/group/condition; each independent experiment included 4-6 animals. ^∗^*p* < 0.05 compared to other three groups. ^#^*p* < 0.05 compared to their vehicle-treated counterparts. ^†^*p* < 0.05 compared to sham-operated groups.

The profile of changes in each ILC subsets and the response to TAT or TAT-GILZ were very similar to those described for experiments using cannabidiol or its vehicle. Briefly, IRI was associated with significant increases in ILC1s and ILC3s but significant reduction in ILC2s; treatment with TAT-GILZ restored the frequency of ILC2s to those of sham control groups while ILC1s and ILC3s were partially restored and remained significantly higher than sham-operated kidneys (Figures 2(a)–2(d)).

**Figure 3:**
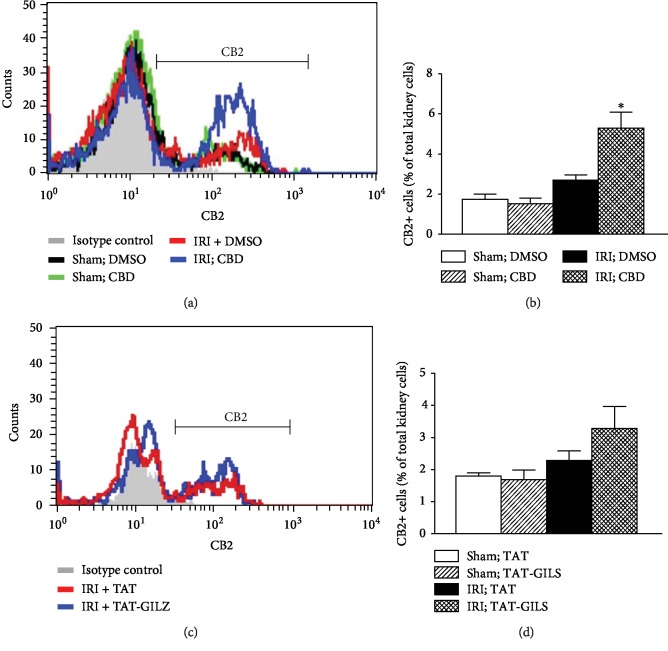
Effects of cannabidiol or GILZ on CB2-positive kidney cells. Kidney cell preparations from experimental groups, as described under Methods, were subjected to flow cytometry analyses for CB2 expression. (a, c) Representative histograms while bar graphs (b, d) show quantitative data for CB2-positive cells for experimental groups. Data are means ± SEM, 4-5 animals (CBD and vehicle conditions) and 6 animals (TAT and TAT-GILZ conditions) for each group; each independent experiment included 3-6 animals. ^∗^*p* < 0.05 compared to other three groups.

We next determined whether treatment with cannabidiol or TAT-GILZ affects expression of CB2 in whole kidney cell preparations. Induction of IRI did not affect frequencies of CB2+ cells (Figure 3). However, while treatment with TAT-GILZ did not significantly affect CB2+ cells, treatment with cannabidiol significantly increased CB2+ cells in IRI kidneys compared to their TAT-treated and DMSO-treated counterparts, respectively (Figure 3).

**Figure 4:**
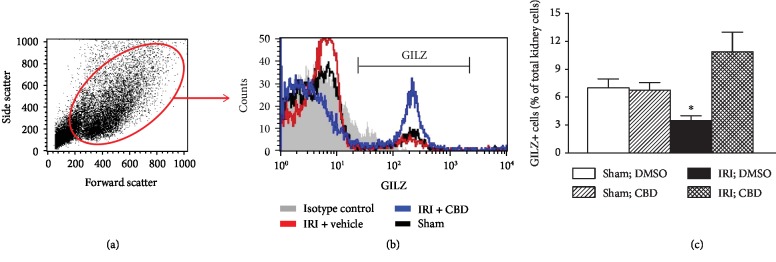
Effects of cannabidiol on GILZ expression in the kidney. Kidney cell preparations from vehicle- and cannabidiol- (CBD-) treated animals, as described under Methods, were subjected to flow cytometry analyses for GILZ expression. (a) Side scatter vs. forward scatter followed by histogram showing GILZ-positive cells. (c) Means ± SEM of 3 animals for group/condition; each independent experiment included 3 animals. ^∗^*p* < 0.05 compared to other three groups.

We also determined the effect of cannabidiol on GILZ expression in whole-kidney cell preparations. As shown in Figure 4, vehicle- and cannabidiol-treated sham-operated kidneys displayed similar frequencies of GILZ+ cells. However, induction of IRI significantly reduced frequencies of GILZ+ cells in vehicle-treated kidneys, an effect abrogated in cannabidiol-treated IRI kidney (Figure 4(c)).

**Figure 5:**
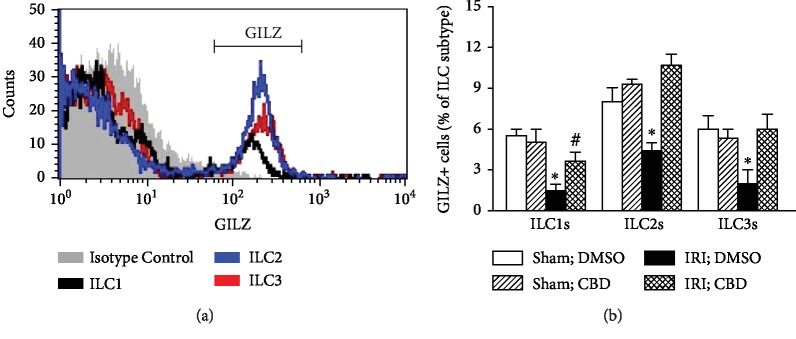
Effects of cannabidiol on GILZ-positive ILC subsets. Kidney cell preparations from vehicle- and cannabidiol- (CBD-) treated animals, as described under Methods, were subjected to flow cytometry analyses for GILZ expression by each subset of ILCs. (a) Histogram showing GILZ-positive cells; (b) means ± SEM of 3 animals for each group/condition; each independent experiment included 3 animals. ^∗^*p* < 0.05 compared to other three groups for each ILC subset. ^#^*p* < 0.05 compared to DMSO-treated sham kidneys for ILC1s.

We next determined expression of GILZ in ILCs and whether treatment with cannabidiol affects each subset of ILCs which also express GILZ. Cannabidiol treatment did not affect GILZ+ subsets of ILCs, expressed as percent of each ILC subtype, in sham-operated kidneys. However, induction of IRI reduced each subset of GILZ+ ILCs (Figure 5). Treatment of IRI kidneys with cannabidiol significantly increased each subset of GILZ+ ILCs but the effect was more marked for ILC2s and ILC3s, achieving values similar to those of their sham-operated counterparts (Figure 5).

**Figure 6:**
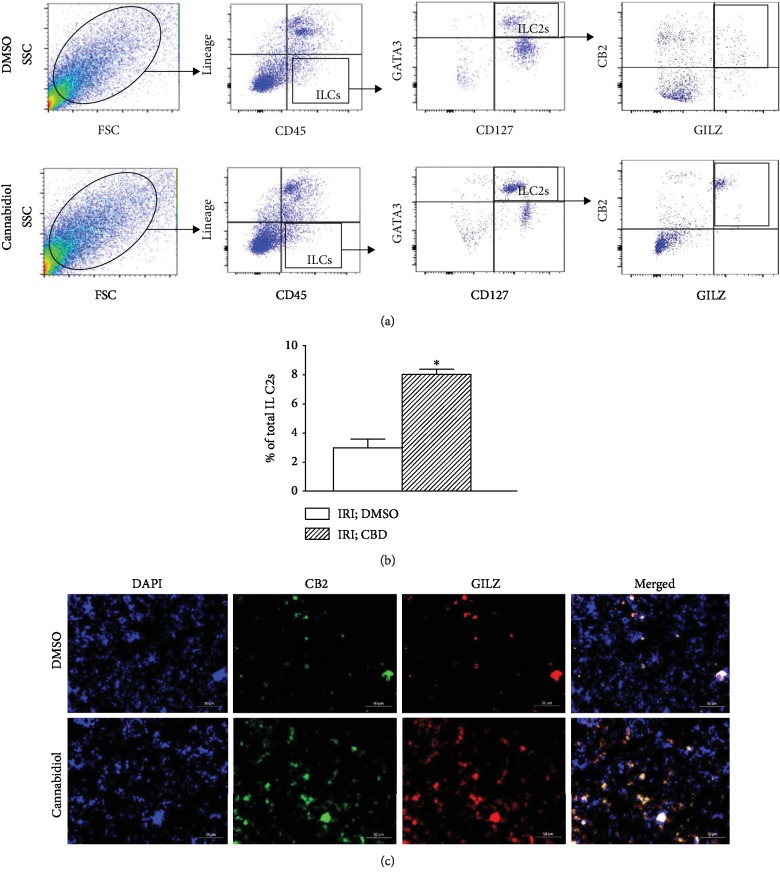
Effects of cannabidiol on CB2 and GILZ expression in ILC2s. Cell preparations from ischemic-reperfused kidneys of vehicle- (DMSO-) and cannabidiol- (CBD-) treated mice were initially sorted for ILC2s, as described in Methods, followed by their identification as CB2+, GILZ+ ILC2s using both analytical flow cytometry (a, b) and immunofluorescence imaging (c); (b) quantitative data show means ± SEM of *n* = 3-4 animals/group. ^∗^*p* < 0.05 compared to the other group.

In light of the more profound impact of cannabidiol on GILZ+ ILC2s (Figure 5) and the demonstration that the treatment also increases CB2+ cells in whole-kidney cell preparations (Figure 3), we next determined whether cannabidiol treatment increases expression of both CB2 and GILZ in ILC2s; these studies used flow cytometry and immunofluorescent protocols. As shown in Figures 6(a) and 6(b), compared to vehicle treatment, cannabidiol treatment significantly increased the percent of ILC2s which was positive for both CB2 and GILZ in the ischemic-reperfused kidney, an effect also evident from merged immunofluorescent images shown in Figure 6(c).

## 4. Discussion

The present study shows that (a) IRI affects the profile of ILC subsets in the kidney; (b) cannabidiol and GILZ exert similar effects on ILC subsets, restoring their frequencies towards those of sham-operated kidneys; (c) cannabidiol increases both CB2+ and GILZ+ cells in the kidney subjected to IRI; (d) IRI reduces frequencies of GILZ+ ILCs, an effect less prominent for ILC2s; (e) cannabidiol restores GILZ+ ILC subsets in the kidney subject to IRI, an effect most marked for ILC2s but less prominent for ILC1s; and (f) cannabidiol increases CB2+ GILZ+ ILC2s in the IRI kidney. Collectively, these novel observations indicate that both cannabidiol and GILZ are major regulators of ILC subtypes and that cannabidiol, likely via a mechanism dependent on GILZ modulation, promotes the suppressive/protective ILC2s in acute kidney injury.

Utilizing the murine model of AKI, simulated by renal IRI, we now show significant increases in ILC1s and ILC3s but a significant reduction in ILC2s in the damaged kidneys compared to their sham-operated counterparts; these changes should be conducive to a proinflammatory environment in the damaged kidney. To our knowledge, this is the first study which demonstrates the profile of ILC subsets in AKI. Previous studies have focused on therapeutic modulation of type 2 immunity, via maneuvers to amplify ILC2s, on the outcome of AKI [22]. Huang et al. [31] showed that Il-25-treated mice subjected to renal IRI display increased serum levels of IL-4, IL-5, and IL-13 in association with increased frequencies of ILC2s and improved renal function. Further, adoptive transfer of ILC2s reduced renal functional and histopathological features in IRI mice accompanied with induction of suppressive/regulatory macrophages in the kidney. Further, Cao et al. [32] have shown that treatment with recombinant IL-33 increases serum and kidney levels of IL-4 and IL-13 along with increased frequency of ILC2s (as well as regulatory macrophages and T cells) in mice kidney subjected to IRI; these changes were associated with reduced renal structural injury and functional abnormalities and improved survival of mice subjected to AKI. Further, authors showed that adoptive transfer of ILC2s also protects the kidney against IRI. Authors concluded that activation IL-33-ILC2 axis represents a therapeutic strategy to confer renoprotection in AKI. Others have shown that in adriamycin-induced glomerulonephritis, a model of chronic proteinuric renal injury, treatment of animals with IL-33 improved the course of the disease via an ILC2-mediated induction of protective type 2 response; the treatment also increased IL-5 which promoted accumulation of eosinophils in the kidney and ameliorated glomerulonephritis [23]. A more recent study investigated tissue localization of ILC2s in the normal kidney and whether their deficiency is of consequence for the kidney subjected to IRI [33]. Authors show that ILC2s are located in close proximity to the renal vasculature and constitutively express high levels of IL-5 under hemostatic conditions. Further, they show that deficiency or depletion of ILC2s in animals subjected to renal IRI does not affect histopathology, collagen deposition, or mRNA expression of injury-associated (*Lcn2*), inflammatory (*Cxcl1*, *Cxcl2*, and *Tnf*) or extracellular matrix (*Col1a1*, *Fn1*) factors. These observations led authors to conclude the potential for ILC2 redundancy and that other components of type 2-mediated immunity likely compensate for loss of ILC2s thereby accounting for lack of exacerbated kidney injury under these conditions [33].

While the focus of recent research has been to unravel the role of ILC2s in pathogenesis of AKI, our demonstration of marked increases in ILC1s and ILC3s in this condition is suggestive of their pathogenic roles in determining the outcome of renal IRI. Thus, there is a need to identify therapeutic options that can modulate ILC subsets to those of the normal kidney with the objective of conferring beneficial impact to the ischemic-reperfused kidney. In light of our recent demonstration that both cannabidiol and GILZ exert significant renoprotective effects, as reflected by reduction in cell death and improved functional outcomes, in association with similar changes in polarization of neutrophils and T lymphocytes in favor of their regulatory/suppressive phenotypes [16, 17], we sought to establish the impact of each agent on the profile of ILC subsets of the kidney subjected to IRI. Interestingly, cannabidiol and GILZ caused similar changes in the profile of ILC subsets in the ischemic-reperfused kidney, restoring their levels towards those of sham-operated kidneys. However, while cannabidiol and GILZ normalized ILC2s of ischemic-reperfused kidneys, ILC1s and ILC3s remained significantly higher in the ischemic-reperfused kidneys compared to their sham-operated counterparts. These novel observations are suggestive of potential crosstalk between cannabidiol and GILZ in determining frequencies of ILC subtypes in AKI, an aspect further explored as described below.

Earlier studies have suggested an interaction between endocannabinoid and glucocorticoids signaling pathways as evidenced by high expression of type 1 cannabinoid receptor (CB1) in brain regions that are implicated in actions of glucocorticoids [8]. Further, glucocorticoids are shown to mobilize the endocannabinoid system and a functional endocannabinoid system is required for many glucocorticoid-mediated effects [8]. Given that both glucocorticoids and endocannabinoid systems exert context-specific regulatory/suppressive roles in the immune system adds further credence to functional crosstalk between the two systems although exact mechanisms remain elusive [8]. It is now well-established that GILZ is a pivotal regulator of anti-inflammatory effects of glucocorticoids [5–7]. On the other hand, the effects of endocannabinoids (and exogenous cannabinoids) in immune-/inflammatory-mediated processes have been attributed to both receptor-mediated and nonreceptor-mediated events [8–14]. Nonetheless, it is known that immune cells express CB2 while CB1 is primarily expressed in the brain [8]. Thus, as an initial attempt to investigate an interaction between GILZ and cannabidiol in regulation of ILC subtypes, we determined the frequencies of CB2+ and GILZ+ kidney cells using whole-kidney cell preparations from experimental groups. The results show that while both cannabidiol and GILZ increase frequencies of CB2+ cells in ischemic-reperfused kidneys, the effect is more marked and significant for cannabidiol. This is a rather intriguing observation since ligand exposure usually downregulates receptor expression, an aspect which requires further investigation. Nonetheless, it is noteworthy that while downregulation of brain CB1 is reported following repeated agonist treatment, CB1 receptor density increases in lymphocytes of daily cannabis users [34]. Importantly, frequency of GILZ+ cells was markedly reduced in kidneys subjected to IRI, an effect abrogated with cannabidiol treatment. In light of these observations, we next determined whether cannabidiol treatment affects the proportion of each subset of ILCs which also express GILZ. Indeed, both IRI and cannabidiol exerted profound effects on GILZ+ subsets. First, IRI significantly reduced GILZ+ ILC1s-ILC3s. Second, treatment with cannabidiol restored each subset of GILZ+ ILCs towards those of their sham-operated counterparts with the effect more prominent for ILC2s; however, while cannabidiol also increased GILZ+ ILC1s in IRI kidneys, a significant differential persisted compared to vehicle-treated sham group. To our knowledge, this is the first demonstration of GILZ expression in ILCs and the ability of both IRI and cannabidiol to regulate fractions of GILZ+ ILC subsets. Given the profound impact of cannabidiol on GILZ+ ILC2s and our demonstration that the treatment also increases frequencies of CB2+ cells in ischemic-reperfused kidneys, we sought to determine whether cannabidiol affects expression of both CB2 and GILZ in ILC2s. Indeed, the results show that cannabidiol treatment increases CB2+ GILZ+ ILC2s of ischemic-reperfused kidney, further substantiating our working hypothesis of a functional crosstalk between CB2 and GILZ in regulation of ILCs. Nonetheless, it is noteworthy that while we have focused on the effects of cannabidiol in the context of CB2, a synergistic effect between CB1 and CB2 has been described in relation to analgesia [35]. Thus, an interaction between these receptor subtypes in regulation of ILCs cannot be ruled out. Importantly, while we have focused on studying ILCs, our recent demonstration that cannabidiol and GILZ exert similar effects on polarization of both neutrophils and T lymphocytes [16, 17] raises the possibility of similar crosstalk between cannabidiol and GILZ in regulation of regulatory/suppressive phenotypes of cells of both innate and adaptive immunity. Collectively, our observations provide a plausible explanation for previously reported similarities between the endocannabinoid system and glucocorticoids in regulation of immune and inflammatory processes [8].

In conclusion, the present study reveals important roles for IRI, as well as cannabidiol and GILZ, and their interaction, in regulation of subtypes of ILCs. Our observations substantiate the notion of the remarkable plasticity of these largely tissue resident cells (e.g., ILC2s) to respond and adapt to environmental cues [21]. The emerging role of ILCs as archetypal cells of the immune system, with functional links to other cells of the immune system [19], makes it imperative to unravel molecular determinants of their subtypes and their modulation to beneficially impact disease outcomes (e.g., AKI). Importantly, the demonstration that GILZ likely mediates the impact of cannabidiol in regulation of ILC subtypes (and possibility other immune cells) raises the prospects of targeting GILZ (e.g., via small molecules) to regulate development of immune cell phenotypes in a context- and disease-specific manner.

## Data Availability

The authors confirm that the data supporting the findings of this study are available within the article.

## Abbreviations

AKI: Acute kidney injury
AHR: Aryl hydrocarbon receptor
CBD: Cannabidiol
CB1: Cannabinoid receptor subtype 1
CB2: Cannabinoid receptor subtype 2
DAPI: 4′,6-Diamidino-2-phenylindole
DMSO: Dimethyl sulfoxide
GATA3: GATA binding protein 3
GILZ: Glucocorticoid-induced leucine zipper
GST: Glutathione S-transferase
ILCs: Innate lymphoid cells
IFN*γ*: Interferon *γ*
IL: Interleukin
IRI: Ischemic-reperfusion injury
Lin: Lineage
PBS: Phosphate buffer saline
ROR*α*: RAR-related orphan receptor *α*
ROR*γ*t: RAR-related orphan receptor *γ*t
T-bet: T-box transcription factor
TAT: Transactivator of transcription.
